# The Relationship between Auditory Sensory Gating and Cognitive Functions on Auditory and Visual Modalities in Primary School Children

**Published:** 2019

**Authors:** Rasool PANAHI, Farnoush JAROLLAHI, Mehdi AKBARI, Malahat AKBARFAHIMI, Hamid HAGHANI

**Affiliations:** 1 Department of Audiology, School of Rehabilitation Sciences, Iran University of Medical Sciences, Tehran, Iran; 2 Department of Occupational Therapy, School of Rehabilitation Sciences, Iran University of Medical Sciences, Tehran, Iran; 3 Department of Biostatistics, School of Public Health, Iran University of Medical Sciences, Tehran, Iran

**Keywords:** Children, Event-related potentials, Sensory gating, Working memory, Selective attention

## Abstract

**Objectives:**

Considering the common neurological origins, there is a relationship between the sensory gating and cognitive functions. However, there is no adequate information on this issue. In this study, auditory event-related potentials and the sensory gating performance were assessed in P50, N100 and P200 waves. Besides, their relationship with cognitive performance in auditory and visual modalities was investigated.

**Materials & Methods:**

Nineteen normal primary school students (14 boys) were tested in Tehran, Iran from 2017 to 2018. In the auditory modality, the Persian version of the non-word repetition test and monaural selective auditory attention test (mSAAT) were used for assessment of the working memory and selective attention, respectively. In order to evaluate the visual working memory and visual selective attention, Rey-Osterrieth complex figure, selective and divided attention test were used, respectively. A 32-channel EEG system was used for electrophysiological assessment.

**Results:**

The P50 sensory gating was negatively correlated with the visual selective attention (*P*=0.034, r=-0.49) and N100 sensory gating was negatively correlated with the auditory working memory (*P*=0.043, r=-0.48) as well as visual selective attention (*P*=0.039, r=-0.47). For P200, there was a significant negative relationship with auditory selective attention in the right ear (*P*=0.034, r=-0.49).

**Conclusion:**

Sensory gating in children is not a modality-specific phenomenon. Sensory gating in a modality could be associated with cognitive functions in other modalities.

## Introduction

With presentation of a transient acoustic stimulus (S1) to a normal person, a positive auditory event-related potential (ERP) can be detected in the frontocentral region of the skull surface within a time interval of about 50 milliseconds (ms) called P50. If a similar acoustic stimulus (S2) is provided within a short time after the first transient acoustic stimulus, the responded evoked potential it is smaller and hence is inhibited or gated. Reduction of the evoked response to S2 represents the sensory gating performance called P50 sensory gating response (1). In other words, the ability of the brain networks to control response to irrelevant environmental stimuli is called sensory gating. This mechanism protects the brain from the overflow of the information (2). The sensory gating ratio can be defined as the ratio of S2 response amplitude to S1 response amplitude multiplied by 100 (S2/S1*100). Lower ratios reflect better gating capability and stronger reduction of response to irrelevant stimuli. It represents a precautious inhibitory filter mainly performed by a network including auditory and prefrontal cortices (3). 

The prefrontal cortex has several functions. In addition to sensory gating, prefrontal cortex plays an important role in working memory functions and can modulate sensory processing of the brain in regions related to selective attention (4, 5). Considering the common neurological origin and because inhibition of unnecessary input is one of the prerequisites for efficient cognitive processing, including selective attention and working memory (6), there is a relationship between the sensory gating and cognitive functions. Accordingly, sensory gating can improve cognitive abilities through influencing attention and working memory (7). Individuals with higher working memory capacity have a higher ability to maintain selective attention (8). Their performance is also less affected by auditory distraction during visual-verbal and auditory-verbal tasks (9, 10). In this way, working memory can affect sensory gating in the visual and auditory modalities. However, there are inconsistent reports in studies researched on the potential relationship between cognitive functions and preattentive phase of sensory input processing like P50 gating. For example, relations have been reported between P50 gating and attention, memory and learning (6, 11). In contrast, there are studies that do not confirm the existence of such a connection (12, 13). Multisensory interactions in auditory cortex have been already identified. Thus, visual input could affect auditory processing (14). However, how gating function in one modality is related to the cognitive performance in another modality is not well understood.

In addition to the P50, the N100 and P200 waves recorded at a later time window can also represent the sensory gating characteristics (15, 16). N100 and P200 sensory gating could reflect different mechanisms than those reflected by P50 gating (16). These waves represent a triggering of attention or allocation of attention (17). Study on the sensory gating performance at P50, N100 and P200 potentials can represent biological infrastructures that maintain the integrity of cognitive function by preventing the entry of unrelated information to higher processing stages (7). However, less work has been done on sensory gating performance at processing levels after P50 wave. Among the few existing studies on adults, some have pointed to the relationship between cognitive functions and N100 and P200 gating (7, 16). Limited information is available on the sensory gating performance in children. 

Given the theoretical importance of sensory gating in preserving the integrity of cognitive functions and normal development of children, we aimed to examine the relationship between auditory sensory gating at P50, N100 and P200 waves and auditory/visual selective attention and working memory abilities.

## Materials & Methods

**Subjects**

This cross-sectional study was performed on 19 normal primary school students (14 boys) with a mean age of 9.47 ± 0.71 years. They were selected from the schools on districts 3, 5 and 14 of Tehran City, Iran from 2017 to 2018. All the subjects were right-handed monolingual native Persian speakers. No one had a history of psychiatric or neurological problem, head trauma, previous records of repetitive ear infection or hearing loss. Pure-tone thresholds for all the children were equal or better than 20 dB HL at 250–8000 Hz octave frequencies. 

Informed consent was obtained from all the parents. The study was approved by the Ethics Committee of Iran University of Medical Sciences, Tehran, Iran.

**Cognitive assessments**

Cognitive-behavioral and electrophysiological assessments were carried out in two separate sessions. Behavioral evaluations were conducted in a quiet room in the children’s school and electrophysiological evaluations were conducted at the Audiology Clinic, School of Rehabilitation Sciences, Iran University of Medical Sciences, Tehran, Iran. Cognitive assessments of the two auditory and visual modalities included working memory and selective attention tests. The auditory working memory was evaluated using the Persian version of the non-word repetition test (18). In this test, forty meaningless words were presented to the children with live sound and covered mouth. The children were asked to repeat the conversations with a correct phonological sequence. The assessment of selective auditory attention was done using the Persian version of monaural selective auditory attention test (mSAAT), specifically designed for the assessment of children in the elementary school and its validity and reliability has been determined (19). 

Rey-Osterrieth complex figure test (RCFT) was used to evaluate the performance of visual working memory in children (20). In this test, the subjects were instructed once to draw the RCFT figure carefully, and after 2 min, they were asked to draw again, what they remembered about the figure. The scores were rated by an experienced child occupational therapist. Evaluation of visual selective attention was done using selective and divided attention test software (21) on a laptop. In this test, in the beginning, two target images were presented to the children so that they became familiar with the test objective. During the test, different images were presented at the center of the monitor with a duration of 250 ms and an inter-stimulus interval (ISI) of 1000 ms. Participants were required to press the ‘space’ key whenever they saw any of the target images.

**Electrophysiological assessment**

For ERP recordings, subjects were seated on a comfortable chair in a sound-attenuated and dimly light room. They were asked to look at a monitor that was playing a silent animated movie in a distance of 100 cm. Free field audio stimuli were presented at 70 decibels A through loudspeakers placed next to the left and right sides of the monitor. The acoustic stimuli consisted of 1000 Hz tone bursts with a duration of 30 ms (4 ms rise/fall and 22 ms plateau) (22). Audio stimuli were presented in 2 blocks, each block contains 35 paired tones (70 pairs of stimuli per person). The interval between the two stimuli in a pair of stimuli was equal to 500 ms and the time interval between each pair of stimulus with the next pair was 8 sec (22). Participants were instructed to just watch the movie and not respond to the tones in any way. They were asked to maintain their gaze on the monitor.

Auditory evoked potentials were recorded from the scalp at a sampling rate of 512 Hz using 31 ag/agcl electrodes. An electrocap used includes 29 electrode sites from the 10-10 system (FPz, FP1, FP2, Fz, F3, F4, F7, F8, FCz, FC3, FC4, FT7, FT8 , Cz, C3, C4, C5, C6, T7, T8, TP7, TP8, Pz, P3, P4, O1, O2, A1 and A2) (23), and two electrodes for eye movement control. Horizontal electrooculographic (EOG) signals were recorded using an electrode at the left external canthi, and vertical EOG signals were recorded from an electrode below the left eye. All scalp electrodes, as well as the EOG electrodes were referenced online to A2. The signals were amplified 10000 times with on-line bandpass filter from 0.4 to 200 Hz. All electrode impedances were kept below 20 kΩ during recording (24, 25). Recordings were analyzed offline using the EEGLAB toolbox version 14.1.1b (26) and MATLAB 2014a (The MathWorks, Natick, MA, USA). In the offline analysis, data were low-pass filtered at 46 Hz in order to remove completely the 50 Hz power line noise. All electrodes were referenced mathematically to average earlobes (A1+A2). An independent component analysis (ICA) was performed to remove blink artifacts, heartbeat artifacts, etc. For P50 analysis, data were high-pass filtered at 5 Hz and epoched -100 to +200 ms according to stimulus onset point. The largest positive wave in the latency range of 40 to 85 ms was defined as P50. For N100 and P200 analysis, data were low-pass filtered at 35 Hz and data epoching window was -100 to +400 ms from stimulus onset point. The largest negative wave in the latency range of 90 to 160 ms was defined as N100 and the largest positive wave in the latency range of 140 to 250 ms was defined as P200 (7). The epochs were baseline-corrected concerning the mean voltage of the 100 ms pre-stimulus period. Epochs containing artifacts greater than or equal to 75 µV were rejected (25). If any response was not observed to the S2, it was considered as complete inhibition of response and its amplitude value was assigned as 0.01 µV in the statistical analysis (27). For better demonstration of the results, we performed a grand average to all ERPs. To do so, the ERP waveforms for the separate individuals were simply summed together and then divided by the number of individuals. 

**Statisti** **cs**

The test results were analyzed using SPSS (ver. 16.0, Chicago, IL, USA). Descriptive statistics were presented as the mean and standard deviation (SD). The Kolmogorov-Smirnov test was used to determine if the sample data have a normal distribution. Analytic statistics were done using Pearson’s correlation and independent samples t-test. A *P*-value of <0.05 was determined to be statistically significant.

## Results

P50, N100, and P200 waves were identified for the S1 in all of the children. For the S2, the N100 and P200 waves were not detected in one person, considered as a complete suppression of the response and a value of 0.01 μV was considered for it. Among all of the electrode sites, the response was better detectable in the Cz and this electrode location was used for further analysis. The Amplitude for each wave was defined based on its preceding peak or trough. The average scores for cognitive tests and the average amplitude and latency of the P50, N100 and P200 waves and the measurements related to the sensory gating are listed in Tables 1 and 2.

**Table 1:** Mean±SD of cognitive tests (n=19)

Test	RCFT	Visual attention	Non-word	mSAAT (Right)	mSAAT (Left)
Mean±SD	0.08±2.4	0.85±0.08	0.91±0.04	0.86±0.04	0.88±0.05

RCFT: Rey-Osterrieth complex figure test

**Table 2:** Mean±SD of recorded ERPs (n=19)

	P50		N100		P200	
Stimulus	S1	S2	S1	S2	S1	S2
Amplitude (µV)	3.27±1.7	1.70±1.1	6.66±3.5	2.71±2.9	7.78±4.8	3.52±2.5
Latency (ms)	60.44±7.5	55.87±14.9	99.67±15	99.18±18.4	162.97±16.1	159.67±26.7
S2/ S1 (%)	50.4±22.8		35.9±19.1		48.0±21.4	

S1: first stimulus in the paired stimuli – S2: second stimulus in the paired stimuli

The study of the relationship between cognitive abilities in different modalities showed that visual selective attention and auditory working memory had a marginally significant correlation (*P*=0.06, r=0.43). Investigating the relationship between cognitive assessments and the amplitude of the ERPs showed a significant negative relationship between auditory working memory and the amplitude of p50 (*P*=0.006, r=-0.6) and N100 (*P*=0.003, r=-0.64) waves. Moreover, auditory working memory was negatively correlated with the latency of p50 (*P*=0.003, r=-0.64) and N100 (*P*=0.04, r=-0.47). Visual working memory had no significant relationship with any of the waves. 

**Figure 1:**
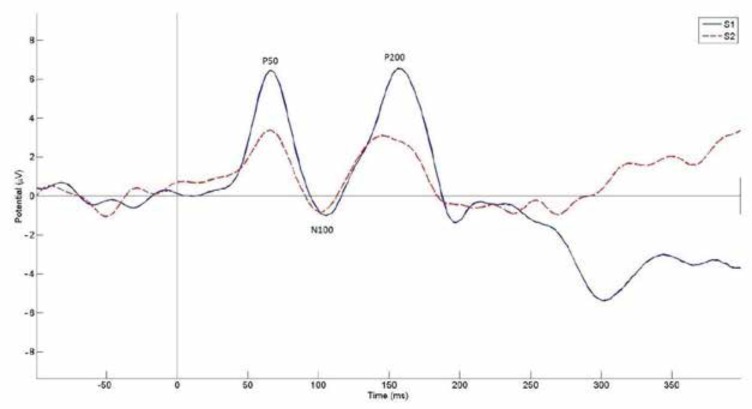
The grand average ERPs for S1 and S2 at Cz

Using paired-samples t-test, the amplitudes for the S1 waves were significantly larger than the amplitude for the S2, which indicates a sensory gating effect for all waves (P50: *P*<0.001, N100: *P*<0.001, P200: *P*<0.001). The assessment of the relationship between sensory gating and cognitive abilities showed that p50 sensory gating was negatively correlated with the visual selective attention (*P*=0.034, r=-0.49), N100 sensory gating was negatively correlated with the auditory working memory (*P*=0.043, r=-0.48) and visual selective attention (*P*=0.039, r=-0.47). For P200 sensory gating, there was a significant negative relationship with auditory selective attention in the right ear (*P*=0.034, r=-0.49). The grand average ERPs for S1 and S2 at Cz is shown in Figure 1.

## Discussion

In the present study, the relationship between working memory and selective attention in the visual and auditory modalities was examined. There was a relatively significant relationship between visual selection and auditory working memory in children. 

Working memory capacity is said to be a tool for measuring cognitive differences between individuals. This association can be attributed to individual differences in the ability to control attention (8, 28). However, little is known on the relationship between these cognitive functions in different modalities. One of the first evidence was provided by investigating the generality of attention control mechanisms associated with working memory (29), which showed a link between auditory attention and visual working memory. Attention and working memory might be considered as overlapping constructs (5). The amount of visual information that a person can store in the visual working memory is predictive of how much that person is able to focus his/her hearing on a particular sound (29). The finding of the present study together with previous reports, suggests supra-modal attention networks that control the flow of information into working memory. This can broaden one’s knowledge on the relationship between cognitive function in different modalities, as this relationship is present at early school years. However, this relationship can become stronger or undergoes some changes because of maturation during adulthood, which needs to be studied. 

In the present study, investigation on the relationship between ERPs and cognitive assessments showed that auditory working memory had a negative correlation with amplitude and latency of P50 and N100. This means that in individuals with better performance at working memory task, ERPs were recorded earlier and with smaller amplitude. Higher working memory capacity is related to smaller N100 amplitude and these individuals have more ability to resist allocating attention in situations in which there is an auditory distractor (30). The attenuation of P50 and N100 amplitudes in association with increased working memory performance can be interpreted according to the information-processing theory, assuming that the P50 is a part of gamma band response synchronization of EEG (31, 32). Since gamma-band activity is associated with synchronized cortical networks involved in attention and working memory (33), P50 and N100 can have common features with attention and working memory operations. This is in accordance with previous report on P50 and N100. Significant negative correlation between P50 and N100 latencies with the working memory scores, suggests that the process of registration of auditory stimuli and orienting subconscious attention towards it might be faster in individuals with higher working memory scores (30, 34).

Our results showed that selective auditory attention in the right ear had a significant relationship with the N100 amplitude. Selective listening is the ability to listen to a stimulus and ignore another stimulus at the same time, as in the cocktail party phenomenon (35). This situation is similar to the situation in the mSAAT test used in the current study. The N100 can reflect the early selective attention in the auditory cortex (30) and that the selective auditory attention can modulate stimulus processing in the auditory cortex in the latency range of 80 to 130 ms (36). The link found here can be related to stronger relationship between the right ear and the left hemisphere. Sensory organs in the right ear are more sensitive to detect a signal in the presence of noise due to the connection with the left hemisphere (37).

Sensory gating in different waves from P50 to P200 can be related to cognitive functions such as selective attention and working memory in both auditory and visual modalities. In most studies, sensory gating evaluations were done on auditory modality, whereas cognitive assessments were conducted on visual modality, which made it difficult to generalize the results (7, 30, 38). The present study is one of the few cases tried to assess selective attention and working memory in both visual and auditory modalities. 

Our findings showed that gating in the P50 wave was sensitive to selective attention and smaller ratios were seen in people who had better performance. The attention model (39) can be helpful in further explanation of this finding. According to this model, the attention process consists of several steps. The first step involves an orienting component that is likely to occur at intervals of less than 150 ms after an event. During this step, a cue will be selected from the sensory inputs to determine the stimulus to be attended. The present findings imply the importance of the role of sensory gating in the early processing of selective attention. Moreover, as noted in some previous studies (27, 38), the results of this study represent the existence of a cross-modality effect on selective attention and sensory gating performance. The observed association with the gating of the P50 wave was only on visual attention and not auditory attention. This can be due to the nature of the mSAAT test, which involves linguistic processing. In terms of the time required for linguistic processing, perhaps the effects of auditory selective attention on linguistic stimulus cannot be seen in the latency of about 50 ms, because the study has shown that the first significant signs of a perceptual distinction between noise and speech in the auditory system are detectable at about 100 ms after the start of the stimulus (40).

The results of our study showed that the N100 sensory gating ratio was significantly smaller in children with better performance on visual selective attention. Such a finding was also obtained for the auditory working memory. Generally, N100 and its sensory gating has been linked to the ability of selective attention and considered as a trigger to allocate attention (7, 17, 41). However, in the present study, in addition to selective attention, this relationship was also observed in working memory. Although a clear relationship has been reported between working memory capacity, attention and N100 wave amplitude (42), to the best of our knowledge, there is no available report on the direct connection between N100 sensory gating and working memory in children. Interpretation of the findings of the present study in the light of previous studies seems difficult. One of the reasons for the difference in the results of the current and that of previous studies might be the study population. Because maturity of the N100 and its biological substructures is slow, it continues until early adulthood (43). Additionally, to record a reliable N100 wave in children under the age of 10, the ISI must be at least 1000 ms or longer (44). Since this ISI value cannot be used in the stimulation pattern associated with sensory gating response, the interpretation of these findings on N100 sensory gating in children is not yet clear.

Sensory gating in the P200 wave had a significant relationship with auditory selective attention. The P200 is one of the earliest signs of a conscious perception of the acoustic stimulus, as well as an early sign of allocation of attention (17). Therefore, the sensory gating in P200 wave will also be associated with attention functions. In this study, such a relationship was observed as expected. Considering the language base of the auditory attention test in the present study, in later waves, the sensory gating will be associated with selective attention performance. However, this connection was seen only in the right ear. Given the strong relationship between the right ear with the left hemisphere of the brain and its role in the processing of speech in the presence of background noise (37), functions related to the allocation of attention and awareness of spoken stimuli are mainly reflected in the right ear.

**In conclusion, **stronger sensory gating is associated with higher cognitive abilities in children and suggests its protective role in cognitive processes. Sensory gating in a modality can be associated with cognitive functions in other modalities; therefore, sensory gating is not a modality-specific phenomenon. More studies are needed to generalize these findings.
